# Two Unrelated 8-Vinyl Reductases Ensure Production of Mature Chlorophylls in Acaryochloris marina

**DOI:** 10.1128/JB.00925-15

**Published:** 2016-04-14

**Authors:** Guangyu E. Chen, Andrew Hitchcock, Philip J. Jackson, Roy R. Chaudhuri, Mark J. Dickman, C. Neil Hunter, Daniel P. Canniffe

**Affiliations:** a Department of Molecular Biology and Biotechnology, University of Sheffield, Sheffield, United Kingdom; b ChELSI Institute, Department of Chemical and Biological Engineering, University of Sheffield, Sheffield, United Kingdom

## Abstract

The major photopigment of the cyanobacterium Acaryochloris marina is chlorophyll *d*, while its direct biosynthetic precursor, chlorophyll *a*, is also present in the cell. These pigments, along with the majority of chlorophylls utilized by oxygenic phototrophs, carry an ethyl group at the C-8 position of the molecule, having undergone reduction of a vinyl group during biosynthesis. Two unrelated classes of 8-vinyl reductase involved in the biosynthesis of chlorophylls are known to exist, BciA and BciB. The genome of Acaryochloris marina contains open reading frames (ORFs) encoding proteins displaying high sequence similarity to BciA or BciB, although they are annotated as genes involved in transcriptional control (*nmrA*) and methanogenesis (*frhB*), respectively. These genes were introduced into an 8-vinyl chlorophyll *a*-producing Δ*bciB* strain of Synechocystis sp. strain PCC 6803, and both were shown to restore synthesis of the pigment with an ethyl group at C-8, demonstrating their activities as 8-vinyl reductases. We propose that *nmrA* and *frhB* be reassigned as *bciA* and *bciB*, respectively; transcript and proteomic analysis of Acaryochloris marina reveal that both *bciA* and *bciB* are expressed and their encoded proteins are present in the cell, possibly in order to ensure that all synthesized chlorophyll pigment carries an ethyl group at C-8. Potential reasons for the presence of two 8-vinyl reductases in this strain, which is unique for cyanobacteria, are discussed.

**IMPORTANCE** The cyanobacterium Acaryochloris marina is the best-studied phototrophic organism that uses chlorophyll *d* for photosynthesis. Unique among cyanobacteria sequenced to date, its genome contains ORFs encoding two unrelated enzymes that catalyze the reduction of the C-8 vinyl group of a precursor molecule to an ethyl group. Carrying a reduced C-8 group may be of particular importance to organisms containing chlorophyll *d*. Plant genomes also contain orthologs of both of these genes; thus, the bacterial progenitor of the chloroplast may also have contained both *bciA* and *bciB*.

## INTRODUCTION

The process of photosynthesis, in which solar energy is converted into chemical potential energy, is reliant upon light-absorbing chlorophyll (Chl) pigments that are incorporated into the antenna complexes of phototrophic organisms. Structural modifications to the tetrapyrrole macrocycle of these Chls, which influence the pigment-pigment and pigment-protein interactions within light-harvesting antenna complexes, are responsible for the specific absorption and energy transfer features of the photosystem (1–3).

**FIG 1:**
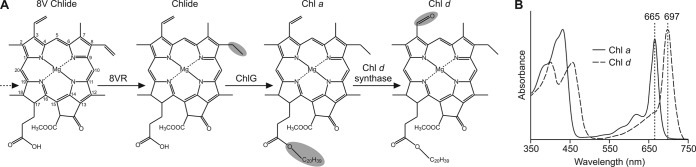
The terminal steps in the biosynthesis of Chls *a* and *d*. (A) The precursor 8V-Chlide (IUPAC numbered) is reduced to Chlide by an 8VR prior to the addition of phytol to the C-17 propionate side chain by Chl synthase (ChlG). In A. marina, the currently unidentified Chl *d* synthase oxidizes the C-3 vinyl group of Chl *a* to a characteristic formyl group. (B) The Chl *d* synthase-catalyzed oxidation results in a red shift in the Q_y_ absorption maximum of the pigment from 665 nm to 697 nm (in methanol).

With the exception of the marine cyanobacterial Prochlorococcus spp. (4), the majority of Chls used by oxygenic phototrophs carry an ethyl group at the C-8 position (8E), the product of an 8-vinyl reductase (8VR) acting on a biosynthetic precursor, 8-vinyl (8V) chlorophyllide (Chlide) (5) (Fig. 1A). Two unrelated classes of 8VR are known to exist in oxygenic phototrophs, BciA and BciB.

BciA was first identified through screening mutants of Arabidopsis thaliana; mutations in the AT5G18660 locus led to the accumulation of 8V- rather than 8E-Chls (6, 7), and recombinant protein produced in Escherichia coli was shown to reduce 8V-Chlide to 8E-Chlide (6). Subsequently, BciA activities were demonstrated for proteins from rice (8), maize and cucumber (9), the green sulfur bacterium Chlorobaculum tepidum (10), and the purple phototrophic bacterium Rhodobacter sphaeroides (11). *In vitro* assays performed with BciA-type 8VRs from various species showed that NADPH is a reductant for this enzyme (8–10, 12).

Although also utilizing 8E-Chls, the genomes of the majority of cyanobacteria do not contain orthologs of *bciA*, indicating the existence of a second, unrelated 8VR. Two studies on the model cyanobacterium Synechocystis sp. strain PCC 6803 (Synechocystis) demonstrated that mutants with mutations in open reading frame (ORF) slr1923 were unable to grow under high light intensities and accumulated 8V-Chl *a* (13, 14). Subsequently, an ortholog of slr1923 from the green sulfur bacterium Chloroherpeton thalassium was shown to complement the Chlorobaculum tepidum bciA mutant, recovering synthesis of 8E-bacteriochlorophyll (BChl) and Chl in this strain, confirming the activity of the second, BciB, class of 8VRs (15). A study on the *in vitro* activity of the BciB-type 8VR from Chloroherpeton thalassium showed that the enzyme is an flavin adenine dinucleotide (FAD)-containing Fe-S protein, deriving electrons from reduced ferredoxin (16).

Acaryochloris marina is the most widely studied organism utilizing Chl *d* for photosynthesis (17–19). Chl *d* differs from Chl *a* in that it carries a formyl group at C-3 rather than a vinyl group (17) (Fig. 1A), and oxygen labeling experiments confirmed that Chl *a* is the direct biosynthetic precursor of Chl *d* (20) (Fig. 1A). The presence of the formyl group red-shifts the Q_y_ absorption band of the unbound pigment by approximately 30 nm compared to that of Chl *a* (Fig. 1B), and Chl *d* was found to account for 92% of the total Chl content of the cell (18). It has also been determined that Chl *d* is used not only for light harvesting as an antenna pigment but also as photochemically active special-pair Chls in both photosystem II (PSII) (21) and PSI (22, 23). The pigment composition of A. marina allows it to efficiently harvest far-red light to drive photosynthesis, an adaptation that permits survival in colonial ascidians (24) and microbial mats (25), where the photosynthetically active radiation is absorbed by the Chl *a* (with or without Chl *b*)-containing phototrophs but far-red light is enriched (26).

While most cyanobacteria utilize BciB to provide reduced Chls for photosynthesis, a small number instead use BciA. Uniquely for cyanobacteria sequenced to date, bioinformatic analysis revealed that the two sequenced genomes of Acaryochloris spp. (A. marina MBIC11017 and Acaryochloris sp. strain CCMEE 5410) contain homologs of both *bciA* and *bciB*. Here we expressed the A. marina genes in a mutant of Synechocystis unable to synthesize 8E-Chl *a* in an attempt to determine whether both ORFs encoded functional 8VRs. Heterologous expression of both genes restored the ability of the strain to grow under high-light conditions and to synthesize reduced Chl *a*. RNA and protein level analyses of A. marina cells demonstrated that both BciA and BciB are present *in vivo*. We hypothesize that two 8VRs are employed to ensure that only Chls carrying 8E groups are synthesized in these strains; possible penalties for the presence of 8V-Chl *d* are discussed.

## MATERIALS AND METHODS

### Bacterial strains and growth conditions.

The bacterial strains and plasmids used in this study are listed in Table 1. E. coli strain JM109 (27) transformed with pPD-FLAG (28) plasmids was grown in a rotary shaker at 37°C in LB medium supplemented with 30 μg · ml^−1^ kanamycin. Synechocystis strains were grown photoautotrophically in a rotary shaker under moderate (50 μmol photons · m^−2^ · s^−1^)- or high (250 μmol photons · m^−2^ · s^−1^)-light conditions at 30°C in liquid BG-11 medium (29) supplemented with 10 mM TES [*N*-tris(hydroxymethyl)methyl-2-aminoethanesulfonic acid], pH 8.2. A. marina was grown photoautotrophically in a rotary shaker under moderate-light conditions (50 μmol photons · m^−2^ · s^−1^) at 28°C in liquid MBG-11 medium (25, 30) supplemented with 10 mM TES, pH 8.2.

**TABLE 1:** Strains and plasmids used in this study

Strain or plasmid	Genotype or characteristics	Source or reference
E. coli JM109	Cloning strain for pPD constructs	Promega
A. marina MBIC11017	WT	R. Blankenship^*a*^
Synechocystis strains		
PCC 6803	WT	R. Sobotka^*b*^
Δ*bciB* mutant	Em^r^ replacement of central portion of slr1923 in WT	11
Δ*bciB*::*nmrA*(Am) mutant	AM1_2394 and Km^r^ replacement of *psbAII* in Δ*bciB* mutant	This study
Δ*bciB*::*frhB*(Am) mutant	AM1_2849 and Km^r^ replacement of *psbAII* in Δ*bciB* mutant	This study
Plasmids		
pPD-FLAG	Cloning site and Km^r^ flanked by *psbAII* up- and downstream regions; Amp^r^	28
pPD[*nmrA*]	AM1_2394 with encoded His_6_ tag cloned into pPD-FLAG (NdeI/BglII)	This study
pPD[*frhB*]	AM1_2849 with encoded His_6_ tag cloned into pPD-FLAG (NdeI/BglII)	This study

a Departments of Biology and Chemistry, Washington University, St. Louis, MO.

b Institute of Microbiology, Department of Phototrophic Microorganisms, Trebon, Czech Republic.

### Construction of Synechocystis mutants containing A. marina genes.

The PCR primers used in this study are listed in Table S1 in the supplemental material. The *frhB* gene was amplified from A. marina MBIC11017 genomic DNA using primers frhBF and frhBR, with the reverse primer encoding a C-terminal hexahistidine tag. The PCR product was digested and cloned into the NdeI/BglII sites of pPD-FLAG vector, and the resulting plasmid was named pPD[*frhB*]. The construction of pPD[*nmrA*] was similar to that described for pPD[*frhB*] except that overlap extension PCR was used to generate full-length *nmrA* containing a silent mutation removing an internal NdeI site found in the native gene. The regions up- and downstream of this restriction site were amplified using the primer pairs nmrA1F/nmrA1R and nmrA2F/nmrA2R, respectively. Primers nmrA1R and nmrA2F were designed to be inversely complementary to each other and did not contain the NdeI site. These amplicons were used as the template for overlap extension PCR with primers nmrA1F and nmrA2R, generating the full-length *nmrA*. The sequenced plasmids were introduced into the Synechocystis Δ*bciB* strain (11). Transformants were selected on solid BG-11 medium containing 10 μg · ml^−1^ kanamycin and fully segregated by incrementally doubling the concentration of antibiotic to 80 μg · ml^−1^. Fully segregated Synechocystis strains were confirmed by colony PCR using primers pPDCheckF and pPDCheckR.

### Extraction and analysis of pigments.

Chls were extracted from Synechocystis cell pellets after washing in 20 mM HEPES (pH 7.2) by adding 9 pellet volumes of 0.2% (vol/vol) ammonia in methanol, vortex mixing for 30 s, and incubating on ice for 20 min. The extracts were clarified by centrifugation (15,000 × *g* for 5 min at 4°C), and the supernatants were immediately analyzed on an Agilent 1200 high-pressure liquid chromatography (HPLC) system. Chl *a* species were separated on a Phenomenex Aqua C_18_ reverse-phase column (5-μm particle size, 125-Å pore size, 250 mm by 4.6 mm) using a method modified from that of van Heukelem et al. (31). Solvents A and B were 80:20 (vol/vol) methanol-500 mM ammonium acetate and 80:20 (vol/vol) methanol-acetone, respectively. Pigments were eluted at 1 ml · min^−1^ at 40°C on a linear gradient of 92 to 94% solvent B over 25 min, increasing to 100% to wash the column. Elution of Chl *a* species was monitored by checking absorbance at 665 nm.

### RNA isolation and RT-PCR analysis.

Total A. marina RNA was isolated from a culture at mid-exponential growth phase using the hot TRIzol method (32) and subsequently treated with the Turbo DNA-free kit (Ambion). The cDNA synthesis and PCR amplification were performed in a single reaction with gene-specific primers using the MyTaq one-step reverse transcription-PCR (RT-PCR) kit (Bioline) according to the manufacturer's instructions. Primer pairs RTnmrAF/RTnmrAR, RTfrhBF/RTfrhBR, and RTrnpBF/RTrnpBR (see Table S1 in the supplemental material) were used to detect transcripts of *nmrA*, *frhB*, and the reference gene *rnpB*, respectively. One hundred nanograms of purified RNA was used in a 50-μl reaction mixture, and the thermal cycling conditions were as follows: 30 min at 45°C, 2 min at 95°C, and then 30 cycles of 10 s at 95°C, 10 s at 60°C, and 30 s at 72°C. No-RT controls were included for each sample by omitting the reverse transcriptase from the reaction mixture. Ten microliters of each PCR product was separated on a 2% agarose gel and visualized by staining with ethidium bromide.

### Proteomic analysis of A. marina.

A cell pellet from 1 ml of culture (*A*_750_ = 0.62) was resuspended in 50 μl 2% SDS–60 mM dithiothreitol (DTT) and mixed with an equal volume of silicon carbide beads (1 mm; BioSpec). The cells were incubated at 95°C for 90 s and then vortexed for 30 s. This treatment was repeated 3 more times before centrifugation at 12,000 × *g* for2 min. The supernatant was transferred to a new vial, the beads were washed with 50 μl 2% SDS–60 mM DTT, and the supernatants were pooled. Total cell lysate protein was precipitated, dissolved in 8 M urea, reduced, S-alkylated, and digested with a combination of endoproteinase LysC and trypsin according to the method of Zhang et al. (33). Two micrograms of each proteolytic enzyme was used based on a 1:25 (wt/wt) enzyme/substrate ratio, as estimated by the method of Kalb and Bernlohr (34). After digestion, trifluoroacetic acid (TFA) was added to a final concentration of 0.5%, the peptide fragments desalted using a C_18_ spin column (Thermo Scientific), and the eluate dried by vacuum centrifugation. The sample was redissolved in 0.1% TFA–3% acetonitrile, and 450 ng was analyzed in duplicate by nanoflow liquid chromatography (Dionex Ultimate 3000 RSLCnano; Thermo Scientific) coupled to a mass spectrometer (MS) (Q Exactive HF Orbitrap; Thermo Scientific) operating with data-dependent acquisition of 10 tandem MS (MS/MS) scans per MS scan, all in centroid mode. Resolution settings for MS and MS/MS scans were 120,000 and 30,000, respectively, with automatic gain control targets of 1e6 and 1e5 to a 60-ms maximum. Peptides were separated using a 75-min gradient from 97% solvent A (0.1% formic acid) to 50% solvent B (0.08% formic acid, 80% acetonitrile). Mass spectra were converted to Mascot generic files using MSConvert (www.proteowizard.sourceforge.net). Protein identification was carried out by searching against the A. marina proteome database (strain MBIC 11017; release date, 3 October 2015; 8,172 entries) (www.uniprot.org/proteomes/UP000000268) using Mascot Daemon v. 2.5.1 running with Mascot Server v. 2.5 (Matrix Science), merging both duplicates into a single search, specifying trypsin as the enzyme in the search parameters and allowing for one missed cleavage. *S*-Carbamidomethyl-cysteine and methionine oxidation were selected as fixed and variable modifications, respectively. MS and MS/MS tolerances were set to 0.01 Da and false-discovery rates determined by searching a decoy database composed of reversed protein sequences.

### Phylogenetic analysis of BciA and BciB.

To investigate the evolutionary context of A. marina BciA and BciB, homologs were identified in the species listed in Table S2 in the supplemental material through the use of the A. marina protein sequences as the query in blastp searches of the predicted proteomes or in tblastn searches of the genome assemblies where gene annotations were not available. Amino acid alignments were generated for BciA and BciB using MUSCLE (35) with default settings, and phylogenies were obtained with RAxML (36) version 8.2.4, using the automated protein model assignment algorithm and a gamma model of rate heterogeneity (-m PROTGAMMAAUTO). For comparative purposes, the 16S rRNA sequences of the same organisms were obtained from the SILVA database, and a nucleotide alignment was constructed using MUSCLE with default settings. 16S rRNA phylogenies were obtained with RAxML using the general time-reversible model of nucleotide substitution with the CAT approximation of rate heterogeneity (-m GTRCAT).

### Data set accession numbers.

The complete data set has been deposited to the ProteomeXchange Consortium via the PRIDE partner repository (http://proteomecentral.proteomexchange.org) with the identifiers PXD003139 and 10.6019/PXD003139.

## RESULTS

### Expression of candidate 8VR-encoding genes from A. marina in a Synechocystis Δ*bciB* mutant.

**FIG 2:**
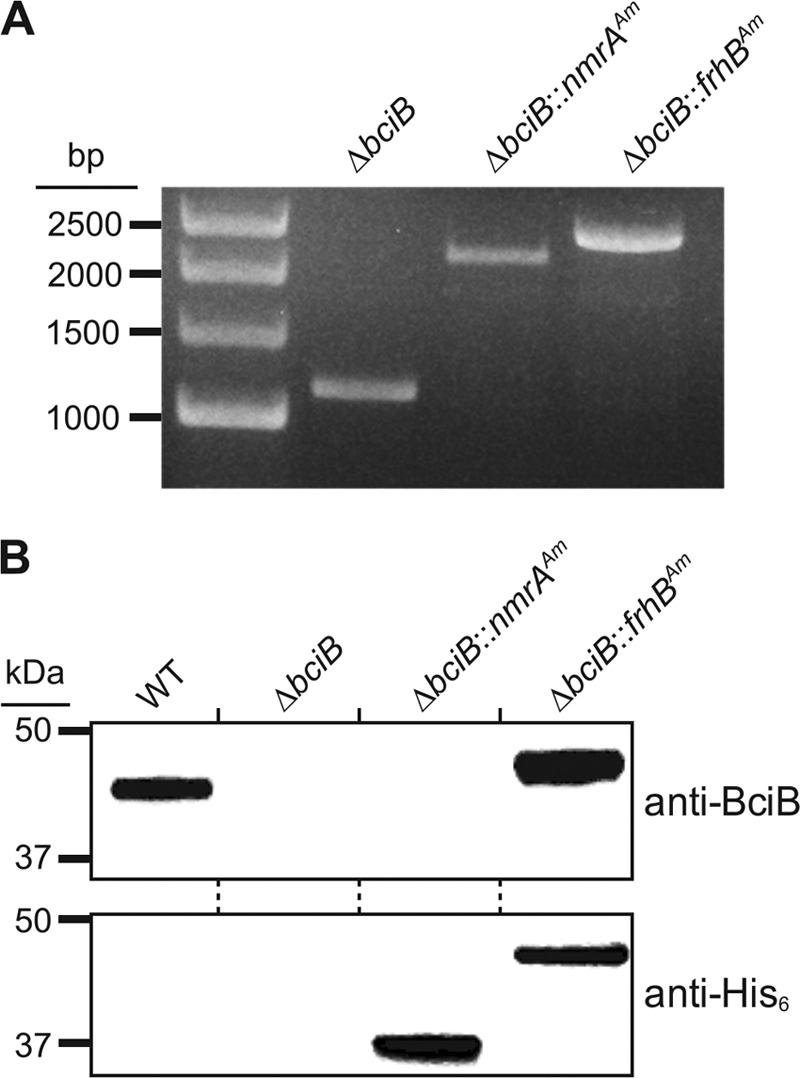
Construction of Synechocystis strains designed to express putative A. marina 8VR-encoding genes. (A) Isolation of fully segregated Synechocystis Δ*bciB* strains containing genes from A. marina, confirmed by colony PCR amplifying the *psbAII* locus. (B) Expression of recombinant proteins was confirmed by resolving membrane fractions from the described strains by SDS-PAGE, transferring to a membrane, and probing with anti-BciB and anti-His_6_ antibodies.

ORFs encoding proteins with high sequence similarity to both BciA and BciB found in the genome sequence of A. marina (26) are annotated as genes encoding a transcriptional regulator (*nmrA*) and a subunit of a NiFe hydrogenase, responsible for coenzyme F_420_ reduction in archaeal methanogenesis (*frhB*), respectively. In order to test the possible 8VR activities of the encoded proteins, *nmrA* and *frhB* were expressed in a Δ*bciB* mutant of Synechocystis (11) that is unable to synthesize 8E-Chl *a* and, as a consequence, is unable to grow under high-light conditions (13, 14). The A. marina genes were integrated into the genome of Synechocystis Δ*bciB* in place of the *psbAII* gene (one of three ORFs encoding the D1 protein of photosystem II; deletion of a single copy of the gene does not affect photosynthetic capability [37]) (Fig. 2A). To determine whether the recombinant proteins were produced in Synechocystis, samples from cultures of wild-type (WT), Δ*bciB*, Δ*bciB*::*nmrA*(Am) and Δ*bciB*::*frhB*(Am) strains, grown under moderate light intensity, were disrupted by bead beating, and the soluble and membrane fractions were separated by centrifugation. The membrane fractions were resolved by SDS-PAGE and transferred to a polyvinylidene difluoride (PVDF) membrane, which was probed with an antibody raised against Synechocystis BciB and, in the absence of an antibody raised against BciA, a commercial anti-His_6_ antibody (Bethyl Laboratories, Inc.) (Fig. 2B). The blot indicates that the recombinant proteins are present, confirming the effective expression of the A. marina genes when under the control of the *psbAII* promoter.

### Functional testing of recombinant proteins.

**FIG 3:**
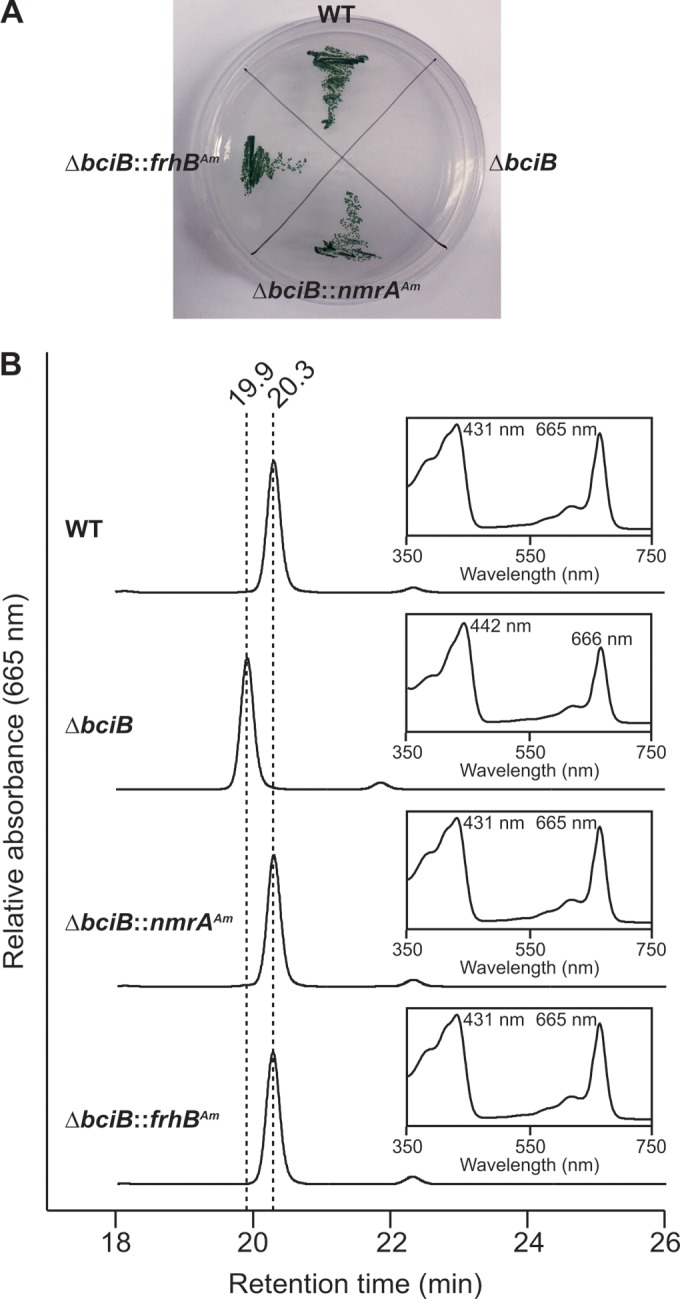
Growth and pigment analysis of described strains of Synechocystis. (A) Strains tested for growth under high light intensity on solid medium. (B) HPLC elution profiles and absorption spectra (inset) of Chls extracted from strains grown under moderate light intensity. Retention times of 20.3 and 19.9 min and Soret absorption maxima at 431 and 442 nm are indicative of 8E-Chl *a* and 8V-Chl *a*, respectively, in the HPLC solvents.

The strains expressing A. marina genes, along with the WT and Δ*bciB* strains, were tested for their ability to grow under high light. Patches of cells were incubated under constant illumination on solid medium. As expected, the Δ*bciB* strain was unable to grow under high light, consistent with previously published results (11, 13, 14), while complementation with both *nmrA* and *frhB* restored the ability of the Δ*bciB* strain to grow under these conditions, comparable to the growth of the WT (Fig. 3A).

Chls from these strains grown in liquid medium under normal light were extracted and analyzed by HPLC (Fig. 3B). The Chl extracted from the Δ*bciB* mutant had a retention time 0.4 min shorter than that of Chl from the WT (Fig. 3B). Analysis of the absorbance profiles of these peaks demonstrated that the Soret band maximum from the Δ*bciB* spectrum is red shifted by 11 nm relative to that from the WT spectrum, indicating that it is the 8V form of the pigment (11, 13, 14). The retention times and absorbance profiles of the Chl peaks from both the Δ*bciB*::*nmrA*(Am) and Δ*bciB*::*frhB*(Am) strains were identical to those of the Chl peaks from the WT (Fig. 3B). Therefore, expression of either *nmrA* or *frhB* successfully recovers the WT status, with respect to 8E-Chl *a* synthesis, and thus we propose that they be reassigned as *bciA* and *bciB*, respectively.

### Identification of 8VR utilized by A. marina.

**FIG 4:**
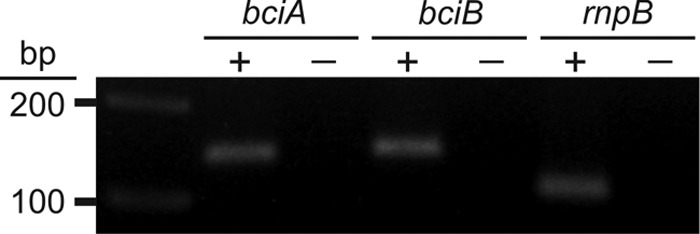
Detection of transcripts of *bciA* and *bciB* in A. marina by RT-PCR. Reactions for *bciA* and *bciB*, along with the *rnpB* housekeeping control, were performed with the inclusion (+) and omission (−) of reverse transcriptase to ensure that samples were not contaminated by genomic DNA.

In order to determine which of the 8VRs A. marina utilizes for Chl biosynthesis or whether both proteins are employed, transcription of *bciA* and *bciB* was checked by RT-PCR, and the presence of the cognate proteins was determined by mass spectrometry. Total A. marina RNA was isolated from a culture at mid-exponential growth phase, treated with DNase to remove any genomic DNA, and used as the template for one-step RT-PCR in which cDNA synthesis and PCR amplification were performed in a single reaction. The housekeeping gene *rnpB*, encoding the RNA subunit of RNase P, was included as positive control. The amplicon generated by RT-PCR displayed a single band with the expected sizes for all the three genes when analyzed by agarose gel electrophoresis (Fig. 4): 140 bp for *bciA*, 142 bp for *bciB*, and 106 bp for *rnpB*. The absence of bands in the no-RT controls eliminated the possibility of genomic DNA contamination. Therefore, we can conclude that both *nmrA* and *frhB* are actively transcribed under the conditions tested in A. marina. Mass spectrometry analysis was performed to verify the presence of NmrA and FrhB proteins in A. marina. Proteins extracted from an A. marina whole-cell lysate were treated with a combination of endoproteinase LysC and trypsin to generate peptide fragments which were then analyzed by nano-liquid chromatography (LC)-MS/MS. Mass spectra, consisting of both peptide ion masses and their product ion profiles, were used as inputs for searching against the A. marina reference proteome database. In total, 1,470 proteins were identified, including both NmrA and FrhB, as shown in Table 2.

**TABLE 2:** Identification of BciA and BciB by proteomic analysis

Protein	Mass (Da)	MOWSE score^*a*^	Sequence coverage (%)	Peptides^*b*^
BciA	36,780	216	27	R.ILVLGGTGTIGR.A, R.ATVAELVK.R, K.FLAEQVFK.N, R.QFYGVVSCLASR.T, R.ESGLIYSIVRPTAYFK.S, K.SVPPGFLNAIATVLGGIAK.I, R.LVDGSEEAERGDFAVF.-
BciB	45,492	58	7	R.TPEEVLAAR.V, R.SVQDSLGLEK.L, R.AGLQTFLETTSR.S

a The two 8VRs were identified by database searching with a *P* value of <0.05 indicating significance, with MOWSE scores representing the inverse of the probability that a match is a random event. The false-discovery rate for this search was 0.75%.

b Tryptic peptides are shown with flanking amino acid residues separated by periods.

### Phylogenetic analysis of BciA and BciB.

**FIG 5:**
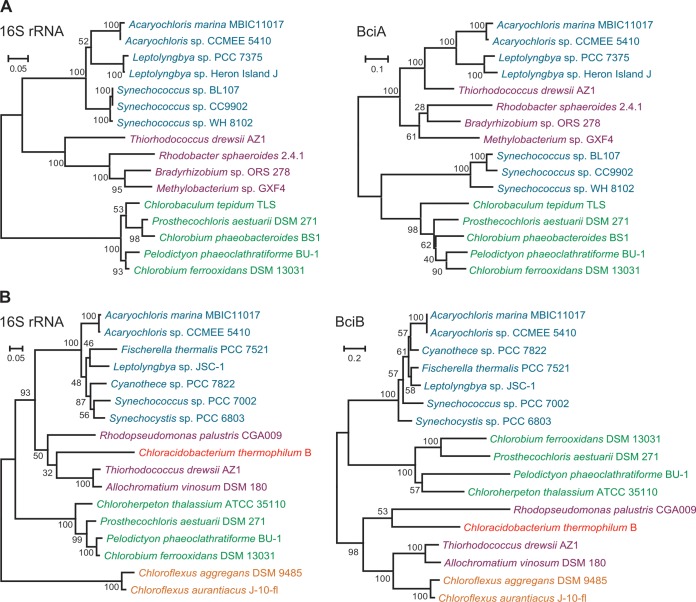
Phylogenetic relationships among 8VR protein sequences compared with parent organism 16S rRNA phylogenies. Maximum-likelihood phylogenies of BciA (A) and BciB (B) homologs, compared with 16S rRNA phylogenies of the same organisms, are shown. The BciA and BciB trees were constructed from amino acid alignments using the PROTGAMMAAUTO model in RAxML version 8.2.4. The rRNA trees were constructed from nucleotide alignments using the GTRCAT model. The numbers on branches indicate the percent bootstrap support from 100 replicates, and the scale bars indicate the specified number of amino acid or nucleotide substitutions per site. Example organisms from cyanobacteria (cyan), purple nonsulfur bacteria (purple), green sulfur bacteria (green), green filamentous bacteria (amber), and Acidobacteria (red) are included.

Comparisons of the phylogenies obtained by maximum-likelihood analysis of BciA and BciB amino acid alignments with those obtained by analysis of 16S rRNA alignments from the same species are shown in Fig. 5A and B, respectively. The phylogenetic positions of A. marina BciA and BciB are both broadly consistent with those shown for A. marina in the 16S rRNA trees, suggesting that the *bciA* and *bciB* genes have not been acquired by horizontal transfer. However, the positions of Synechococcus spp. in the BciA tree and the clade containing the green sulfur bacteria in the BciB tree are inconsistent with the 16S rRNA phylogeny, indicating that there may have been lateral transfer events during the evolution of both *bciA* and *bciB*.

## DISCUSSION

With the current absence of a genetic system for targeted mutagenesis of A. marina, we were unable to determine if the loss of a single 8VR-encoding gene, or loss of 8VR function via disruption of both *nmrA* and *frhB*, would have a negative effect on viability of the cells. Recently, Watabe and coworkers have described the first successful mutagenesis of A. marina cells using a transposon-based system (38); they reported the isolation of a mutant with a transposon insertion mutation in a gene involved in molybdenum cofactor biosynthesis. This mutant could be functionally complemented via introduction of the WT copy of the disrupted gene in *trans*. It is hoped that further development of this method may yield a system for routine targeted mutagenesis in A. marina and other cyanobacteria of interest, allowing the determination of factors involved in far-red-light utilization, including the biosynthesis of Chl *d*. Further, identifying the genes involved in such a process is of significant interest with the recent discovery that some strains of terrestrial cyanobacteria utilizing Chl *a* when grown in white light possess the ability to initiate synthesis of Chls *d* and *f* when cultured in far-red light, coupled with the extensive remodeling of their photosynthetic complexes, a response termed “far-red-light photoacclimation” (FaRLiP) (39).

Loss of 8VR activity in A. marina would result in the production of 8V-Chl *a* and 8V-Chl *d*. 8V-Chl *a* dominates in Prochlorococcus spp. (4) and in a recently isolated strain of the marine eukaryotic protist Alexandrium ostenfeldii (40), and the unreduced form of the pigment is tolerated in plant and cyanobacterial mutants with lesions in 8VR-encoding genes (6, 13, 14). 8V-Chl *d*, however, has yet to be detected in nature. Chl *d* was first reported as a minor pigment in various species of red microalgae (41), although it was later determined that Acaryochloris spp. attached to the surface of the alga were the true source of the pigment (42). Chl *a* can also be readily oxidized to Chl *d* during pigment extraction (43–45). Further, a study by Loughlin et al. determined that vinyl groups of naturally occurring Chls can spontaneously oxidize at C-3, yielding Chl *d*-like pigments, and/or at C-8, yielding novel 8-formyl versions of these Chls (46). The authors measured the Soret/Q_y_ ratios of the substrate and product for each oxidation, comparing the absorption intensity of the high-energy, blue-most-absorbing band of the pigment to that of the lower-energy, red-most-absorbing band. Interestingly, Chls *a*, *d* and *f*, the latter carrying a formyl group at C-2, have Soret/Q_y_ ratios of <1.0, and the ratios of the 8V forms of Chls *a* and *d* are 1.15 and 0.99, respectively. However, the Soret/Q_y_ ratios of both 8-formyl Chl *a* and 8-formyl Chl *d* are 2.34. If the oxidation of the vinyl group at C-3 to yield Chl *d* occurs spontaneously *in vivo* or if the enzyme catalyzing the oxidation is not specific for the C-3 vinyl group, these 8-formyl pigments would be utilized for light harvesting and photochemistry and may result in impaired red-light absorption, thus negating the advantage A. marina holds in its ecological niche conferred by using the far-red-absorbing Chl *d*. This may explain why the two sequenced species in this genus employ two unrelated 8VRs: reduced Chls can be synthesized due to the presence of an alternative enzyme under conditions in which one of the reductants is limiting; e.g., 8V reduction by BciA may dominate when cellular levels of ferredoxin are depleted under iron-limiting conditions. Interestingly, the genomes of the cyanobacterial strains using the FaRLiP response sequenced thus far do not contain multiple copies of 8VR-encoding genes. However, unlike in Acaryochloris spp., Chl *d* is not a dominant pigment, making up only 1 to 2% of the total Chls in the cell (47). We intend to explore the consequences of accumulation of 8V-Chl *d*, and possibly 8-formyl Chl *d*, once the targeted genetic manipulation of A. marina is possible.

The utilization of unrelated enzymes to catalyze a single reaction would not be uncommon in phototrophic organisms. The magnesium protoporphyrin monomethyl ester cyclase and Pchlide oxidoreductase enzymes exist in two distinct classes in oxygenic phototrophs, each employing different reaction mechanisms (48). As with A. marina, many strains of green sulfur bacteria appear to employ multiple 8VRs for (B)Chl biosynthesis, containing either genes encoding enzymes of both classes or more than one copy of *bciB* (15). However, the activities of different conventional 8VRs from the same organism had not been demonstrated until this study. Interestingly, the enzyme catalyzing the first committed step in true BChl biosynthesis in organisms using BChl *a*, Chlide oxidoreductase (COR), is able to use both 8V- and 8E-Chlide substrates, but in each case the product pigment carries an 8E group, demonstrating a surprising additional 8VR activity (49). All known BChl *a*-utilizing phototrophs other than Roseiflexus spp. also contain a *bciA* gene (50). Removal of 8VR function in Rhodobacter sphaeroides, which naturally produces BChl *a*, resulted in the switch to the biosynthesis of BChl *b*, the pigment with the lowest energy-absorbing property of any naturally occurring photopigment (51), leading to the proposal that multiple 8VR activities ensure against the formation of BChl *b* in these organisms. The presence of multiple 8VRs in green sulfur bacteria may also ensure that methylation of the C-8 group is possible; deletion of *bciA* in Chlorobaculum tepidum prevented this methylation and resulted in aberrant assembly of the chlorosome, the specialized light-harvesting antenna in these organisms (52). Similarly, we propose here that Acaryochloris spp. employ two 8VRs to prevent the synthesis of pigments deficient in red/far-red absorption.

The genomes of many plant species, including A. thaliana and rice, which rely on BciA for 8V group reduction, contain orthologs of *bciB* which appeared to have become redundant in these species. However, Meguro et al. demonstrated that the *bciB* ortholog in A. thaliana encodes an enzyme involved in the conversion of Chl *b* back to Chl *a* (53), a process important for greening, acclimation to light intensity, and senescence in higher plants. This enzyme is proposed to have evolved from a diatom BciB and now catalyzes a new step in pigment biosynthesis (53). Of the sequenced cyanobacteria and prochlorophytes, only Acaryochloris spp. appear to contain both *bciA* and *bciB*, and our phylogenetic analysis indicates that neither of the genes was acquired by lateral gene transfer. These observations may provide insights when considering the cyanobacterial progenitor of the chloroplast.

## Supplementary Material

Supplemental material
