# Supplementary material for: Two Unrelated 8-Vinyl Reductases Ensure Production of Mature Chlorophylls in Acaryochloris marina
Source: J Bacteriol. 2016 Apr 14;198(9):1393–400. doi: 10.1128/JB.00925-15 (PMC4836224; doi:10.1128/JB.00925-15)
Supplement: Supplemental material [file supp_198_9_1393__index.html]

Supplemental material 

# Two Unrelated 8-Vinyl Reductases Ensure Production of Mature Chlorophylls in Acaryochloris marina

## Supplemental material

- Supplemental file 1 -

  Tables S1 (Primers) and S2 (Genome sequences)

  PDF, 288K
