# Supplementary material for: Two Unrelated 8-Vinyl Reductases Ensure Production of Mature Chlorophylls in Acaryochloris marina
Source: J Bacteriol. 2016 Apr 14;198(9):1393–400. doi: 10.1128/JB.00925-15 (PMC4836224; doi:10.1128/JB.00925-15)
Supplement: Supplemental material [file JB.00925-15_zjb999094021so1.pdf]

SUPPLEMENTARY MATERIAL

Primer	Sequence (5’-3’)	Restriction site
frhBF	CAAGTACATATGTTGACTATGACTGCGGTACAACCC	*Nde*I
frhBR	CAAGTAAGATCTTTAGTGGTGGTGGTGGTGGTCCGGTAAGTCATATTGACTGAC	*Bgl*II
nmrA1F	CAAGTACATATGATGACTGACGCTAGCACTTCGGA	*Nde*I
nmrA1R	GAGAGAACGTCCATGTGGGCTTGATAGTC	
nmrA2F	GACTATCAAGCCCACATGGACGTTCTCTC	
nmrA2R	CAAGTAAGATCTTTAGTGGTGGTGGTGGTGGAATACAGCGAAGTCGCCCCG	*Bgl*II
pPDCheckF	GAGTAATGGCGTGCAAGGCCCA	
pPDCheckR	TGCTTTATCCGCCGGCACAGG	
RtnmrAF	AGACGCTTCACTGCAGAACA	
RtnmrAR	CATTCAGGAATCCAGGCGGT	
RTfrhBF	ACAGGCATTGTCAGCACCAT	
RTfrhBR	GAGCTGCCAACACCTCTTCT	
RTrnpBF	TGCAAGGGTGCGGTAAGAG	
RTrnpBR	GAGAGCGGGGAAAAGACCAA	

Table S1. List of primers used in this study

Species	GenBank Accession(s)	Assembly status
Acaryochloris sp. CCMEE 5410	AFEJ01000000	Draft
Acaryochloris marina MBIC11017	CP000838-46	Complete
Allochromatium vinosum DSM 180	CP001896-8	Complete
Bradyrhizobium sp. ORS278	CU234118	Complete
Chloracidobacterium thermophilum B	CP002514-5	Complete
Chlorobaculum tepidum TLS	AE006470.1	Complete
Chlorobium ferrooxidans DSM 13031	AASE01000000	Draft
Chloroflexus aggregans DSM 9485	CP001337	Complete
Chloroflexus aurantiacus J-10-fl	CP000909	Complete
Chloroherpeton thalassium ATCC 35110	CP001100	Complete
Cyanothece sp. PCC 7822	CP002198-204	Complete
Fischerella thermalis PCC 7521	AJLL01000000	Draft
Leptolyngbya sp. Heron Island J	AWNH01000000	Draft
Leptolyngbya sp. JSC-1	JMKF01000000	Draft
Leptolyngbya sp. PCC 7375	ALVN01000000	Draft
Methylobacterium sp. GXF4	AKFK01000000	Draft
Pelodictyon phaeoclathratiforme BU-1	CP001110	Complete
Prosthecochloris aestuarii DSM 271	CP001108-9	Complete
Rhodobacter sphaeroides 2.4.1	AKVW01000000	Complete
Rhodopseudomonas palustris CGA009	BX571963-4	Complete
Synechococcus sp. BL107	DS022298	Complete
Synechococcus sp. CC9902	CP000097	Complete
Synechococcus sp. PCC 7002	CP000951-7	Complete
Synechococcus sp. WH 8102	BX548020	Complete
Synechocystis sp. PCC 6803	CP003265-9	Complete
Thiorhodococcus drewsii AZ1	AFWT01000000	Draft

Table S2. List of genome sequences used in this study
